# Nitrogen-mediated improvement of ionic homeostasis and antioxidant capacity enhances rice yield and nitrogen use efficiency under soda saline-alkali stress

**DOI:** 10.3389/fpls.2026.1756775

**Published:** 2026-03-06

**Authors:** Hongyue Wang, Hange Liu, Qingyu Wang, Yang Song, Xudong Wang, Biao Sui, Feng Jin

**Affiliations:** 1 Agronomy College, Jilin Agricultural University, Changchun, China; 2 College of Plant Science, Jilin University, Changchun, China

**Keywords:** nitrogen metabolism enzyme, nitrogen use efficiency, rice, soda saline-alkali, stress physiology

## Abstract

**Background:**

The unique physicochemical properties of soda saline-alkali soils significantly reduce soil nitrogen availability and crop nitrogen use efficiency. While high-yield and high-efficiency cultivation practice offer a key strategy for the synergistic improvement of both crop productivity and resource use efficiency. However, the optimal nitrogen input rate for these practices remains to be determined.

**Methods:**

Hence, a three-year field study was implemented with nitrogen fertilizer application rates ranging from 0 to 325 kg ha^−1^ (0, 125, 175, 225, 275, 325 kg ha^−1^) to assess their effects on ionic balance, stress physiology, nitrogen use efficiency, and grain yield in rice grown under soda saline-alkali soil conditions.

**Results:**

The findings indicate that additional nitrogen fertilizer, particularly at 275 kg ha^−1^ within the high-yield and high-efficiency cultivation practice, significantly reduced the leaf Na^+^/K^+^ ratio and levels of superoxide anion (O_2_^−^) and malondialdehyde (MDA), while increasing K^+^ concentration and enhancing the levels of soluble proteins and proline, as well as the activities of peroxidase (POD), catalase (CAT), and ascorbate peroxidase (APX). Conversely, leaf Na^+^ concentration increased significantly with rising nitrogen application rates. Furthermore, supplementary nitrogen fertilizer significantly improved total nitrogen uptake, nitrogen use efficiency (NUE), and nitrogen agronomic efficiency (NAE) in rice, which can be attributed to markedly enhanced activities of key nitrogen metabolism enzymes. Consequently, grain yields under the N4 (275 kg ha^−1^), N5 (325 kg ha^−1^), N3 (225 kg ha^−1^), N2 (175 kg ha^−1^), and N1 (125 kg ha^−1^) treatments exhibited significant increases of 94.34%, 62.40%, 56.15%, 37.78%, and 24.01%, respectively, relative to the N0 control. These results demonstrate that, within the high-yield and high-efficiency cultivation practice, nitrogen fertilization at a rate of 275 kg ha^−1^ plays a crucial role in improving rice productivity and optimizing nitrogen use efficiency in soda saline-alkali paddy fields.

## 1 Introduction

The Songnen Plain represents one of the world’s three major soda saline-alkali soil distribution zones, containing approximately 2.33 million hectares of soda saline-alkali affected lands with varying degradation levels (Huang et al., 2022). Characterized by dominant sodium carbonate (Na_2_CO_3_) and bicarbonate (NaHCO_3_) components, these soils exhibit elevated soluble salts, exchangeable Na^+^ content, high pH, strong dispersion, poor permeability, and compromised structural stability, presenting significant reclamation challenges (Liu et al., 2024). In addition to ionic toxicity, osmotic stress and oxidative stress, high pH also directly damages crops, causes metabolic flocculation, reduces nutrient effectiveness, delays crop growth, and severely affects yield and quality (Piao et al., 2022; Jin et al., 2024). Rice cultivation has emerged as an effective remediation strategy under adequate water supply conditions, leveraging continuous flooding to mitigate soil structural deterioration and facilitate salt leaching, making it the predominant approach for soda saline-alkali soil reclamation (Xu et al., 2025). However, climate change exacerbates ecosystem vulnerabilities in these areas through persistent salt accumulation, wind erosion of nutrient-deficient sandy substrates, severe freshwater scarcity, and inefficient fertilizer utilization, collectively threatening rice production sustainability.

Soil nitrogen undergoes vigorous nitrification under slightly acidic to neutral conditions, but experiences significant inhibition of nitrification processes and substantial volatile nitrogen losses beyond pH 8.0 (Borchard et al., 2018). Field studies by Zhu et al. (2021) in the Yellow River Delta revealed that although soil salinization delays ammonia volatilization, but ultimately increases cumulative ammonia losses through prolonged emission and enhanced volatilization intensity. Furthermore, soil alkalization indirectly reduces nitrogen availability by altering rhizospheric microbial community structure, notably decreasing *Proteobacteria* abundance by 12-15% and suppressing metabolic pathways related to nitrogen cycling (Wang et al., 2024). Notably, denitrification intensity in salinity-alkaline soils under anaerobic conditions was elevated by 3–5 times compared to conventional soils, facilitating conversion of available nitrogen (NO_3_^-^ and NH_4_^+^) to ineffective molecular N_2_, resulting in nitrogen loss rates of 42-58% (Wang et al., 2024; Teng et al., 2025). Particularly in soda saline-alkali paddy soils, the synergistic effects of elevated pH, strong alkalinity, and high salt content create complex nitrogen transformation pathways, significantly challenging nitrogen effectiveness improvement. In crops, saline-alkali stress significantly inhibits H^+^-ATPase activity, disrupts cellular ion homeostasis and plasma membrane potential, thereby impairing NO_3_^−^ uptake (Wang et al., 2012; Feng et al., 2025). Additionally, the direct competitive effects of HCO_3_^-^, CO_3_^2-^, and Cl^-^ lead to substantial reductions in both NO_3_^-^ content and nitrate reductase (NR) enzyme activity in plants (Li et al., 2022). Studies have demonstrated that saline-alkali stress induces a dramatic decrease in NO_3_^-^ concentration and nitrate reductase gene (*OsNR1*) expression in rice roots, resulting in 55-60% reduction in NH_4_^+^ synthesis and severe ammonium deficiency (Bohara et al., 2018). Furthermore, Wang et al. (2012) revealed that alkaline stress modifies NH_4_^+^ metabolism pathways in rice shoots and leaves, suppressing the activities of key nitrogen metabolism regulatory enzymes glutamine synthetase (GS) and glutamate synthase (GOGAT), while enhancing the glutamate dehydrogenase (GDH) pathway. Thus, developing effective strategies to alleviate soda saline-alkali stress and improve fertilizer efficiency is essential for advancing sustainable agricultural practices in this region.

The application of supplemental nitrogenous fertilizer can mitigate the inhibitory effects of salinity stress on crop nitrogen uptake by maintaining ion balance and mitigating ionic toxicity (Iqbal et al., 2015; Khan et al., 2016), promoting antioxidation and activating enzymes (Rais et al., 2013), increasing the exclusion ability of salt ions through leaves (Javed et al., 2026), while enhancing plant tolerance to saline-alkali stress (Bao et al., 2019; Wang et al., 2023). This approach has been demonstrated to increase crop nitrogen absorption by 15-22%, thereby constituting a crucial strategy for yield improvement in saline-alkaline soils (Teng et al., 2025). However, soda saline-alkaline paddy fields face persistent challenges in nitrogen utilization efficiency due to nitrogen deficiency, poor soil fertility retention, and exacerbated soil salinization caused by excessive fertilizer application (Huang et al., 2022). Furthermore, the traditional rice cultivation practice employing extensive water flooding for “salt leaching and suppression” has been shown to result in 2.3-3.1 times higher nitrogen leaching losses compared to conventional paddy fields (Bao et al., 2019; Xu et al., 2025). Consequently, developing sustainable nitrogen management strategies that synergistically enhance grain productivity, improve nitrogen use efficiency, and minimize fertilizer inputs in saline-sodic paddy systems remains a critical challenge for both agricultural practices and scientific investigations.

Hence, a three-year field experiment was conducted in a soda saline-alkali paddy field to assess the impacts of different nitrogen application rates under a high-efficiency nitrogen management practice on rice ionic balance, stress physiology, nitrogen use efficiency, and grain yield. The objectives of this study were to (i) decode how nitrogen dosage under a high-efficiency management practices modulates cellular stress adaptation mechanisms (ion equilibrium, redox regulation, osmotic adjustment) in saline-alkali cultivated rice, and (ii) systematically assess nitrogen-driven alterations in assimilation-translocation processes, agro-physiological performance, and grain quality attributes under soda-saline edaphic stress.

## 2 Materials and methods

### 2.1 Experimental sites

The three-year field study (2022-2024) was implemented at Jilin Agricultural University’s Soda Salinization Research Station (45°35’N, 123°50’E) in Baicheng City, northeast China. Experimental soils classified as saline-alkaline clay loam (according to International Society of Soil Science taxonomy) exhibited the physicochemical properties shown in **Table 1** for the 0–25 cm layer. This semi-arid region experiences mean annual climatic conditions of 426.2 mm precipitation, 1699.3 mm evaporation, and 4.7 °C temperature.

**Table 1:** Physicochemical properties of the soil.

Soil properties (0–25 cm soil layers)	Value
Sand content (%)	23.13 ± 1.11
Silt content (%)	38.14 ± 1.31
Clay content (%)	37.60 ± 2.09
Bulk density (g cm^−3^)	1.61 ± 0.13
ECe (μS m^-1^)	24.08 ± 0.71
pH	10.10 ± 0.24
SARe (mmolc L^−1^)^1/2^	368.11 ± 4.03
ESP (%)	55.11 ± 2.17
CEC (cmol kg ^-1^)	10.99 ± 0.34
Organic matter (%)	0.64 ± 0.04
Total N (g kg^-1^)	0.27 ± 1.11
Alkali-hydrolysable N (mg kg^-1^)	16.30 ± 1.11
Available P (mg kg^-1^)	9.13 ± 0.68
Available K (mg kg^-1^)	107.25 ± 5.68

ECe, electrical conductivity of soil saturation extract; SARe, sodium adsorption ratio of soil saturation extract; ESP, exchangeable sodium percentage; CEC, cation exchange capacity; N, nitrogen; P, phosphorus; K, potassium.

### 2.2 Experiment design and field managements

The experiment was designed using a randomized block design. A total of six nitrogen fertilizer application rates were established, namely: N0 (non-nitrogen fertilizer, control), N1 (125 kg N ha^−1^), N2 (175 kg N ha^−1^), N3 (225 kg N ha^−1^, local conventional nitrogen level), N4 (275 kg N ha^−1^, local high-yield nitrogen level), and N5 (325 kg N ha^−1^). Each treatment was replicated three times, resulting in a total of 18 experimental plots, with each plot measuring 6.0 m × 5.0 m. Adjacent plots were separated by 0.5 m buffer strips to minimize the risk of cross-contamination. Each plot was equipped with its own irrigation and drainage systems, ensuring independent hydrological management.

Nitrogen fertilizer (N1-N5) field management followed the high-yield and high-efficiency model established in our previous research (Miao et al., 2024). Specifically, the nitrogen fertilizer was applied following a split ratio of 4:2:3:1 for the basal, tillering, panicle initiation, and grain fertilizers (applied at booting stage to enhance photosynthetic capacity and increase grain weight), respectively. For the basal fertilizer, urea (CH_4_N_2_O, 46% N) was applied, while ammonium sulfate ((NH_4_)_2_SO_4_, 21% N) was used for the tillering, panicle initiation, and grain fertilizers. Phosphorus was applied as a basal fertilizer at a rate of 75 kg P_2_O_5_ ha^−1^, while potassium was applied at a total rate of 100 kg K_2_O ha^−1^, with 60% of the application occurring at the basal stage and the remaining 40% at the panicle initiation stage. Phosphorus and potassium fertilizers were applied as single superphosphate (Ca(H_2_PO_4_)_2_·H_2_O, equivalent to 14% P_2_O_5_) and potassium sulfate (K_2_SO_4_, containing 52% K_2_O), respectively. In the N0 treatment, no additional nitrogen was applied; however, the rates and timing of phosphate and potassium applications were aligned with those used in the nitrogen application treatments (N1-N5). In the spring of 2022 (the first year of this experiment), before land preparation, phosphogypsum (CaSO_4_·2H_2_O; 32.90 g·kg^−1^ CaO, 3.29 g·kg^−1^ SiO_2_, 1.97 g·kg^−1^ Fe_2_O_3_, and 1.63 g·kg^−1^ P_2_O_5_; pH 4.19) was incorporated at 10 t·ha^−1^, following local agronomic practices. Additionally, other agronomic management practices, such as pest control measures and irrigation scheduling, adhered to the established local agricultural protocols.

A commercial rice variety, Changbai 9, was used. This variety is saline-alkali resistant, has better yield stability, and shows moderate growth duration. Additionally, the test cultivar was sown in a controlled-environment greenhouse during the second fortnight of April, with subsequent transplantation to field plots (30 × 13.3 cm) completed by the third week of May annually. Individual hills were established with three seedlings per planting unit to maintain optimal population density. Grain maturation was systematically monitored, with harvesting operations initiated within the first ten days of October annually.

### 2.3 Sampling and measurement

#### 2.3.1 Ionic homeostasis parameters

The flag leaves from nine rice plants per treatment (three replicates) were aseptically collected during the physiological maturity phase at heading stage. Plant materials were dehydrated to hygroscopic equilibrium in a forced-air drying oven and then ground into a fine powder. Acid digestion was performed using a nitric-perchloric acid mixture (1:3 v/v) under reflux conditions according to established protocols (Bastías et al., 2004). Quantitative determination of monovalent cations was achieved via atomic emission spectroscopy (Sherwood Model M410 Flame Photometer) with wavelength-specific detection: Na^+^ (589 nm) and K^+^ (766 nm). The Na^+^/K^+^ ratio was computationally derived from molar concentration ratios.

#### 2.3.2 Relevant osmotic regulatory substances and protective enzymes in leaves

The representative flag leaf samples were harvested at heading stage and cryopreserved in liquid nitrogen within 30 s post-excision, followed by ultra-low temperature storage (−80°C) until biochemical characterization. The relevant osmotic regulatory substances and protective enzymes in leaves were analyzed through standardized spectrophotometric protocols. Soluble sugar was quantified via phenol-sulfuric acid chromogenic reaction (glucose calibration curve; the absorbance at 490 nm) following Pistocchi et al. (1997). The soluble protein was determined using Coomassie Brilliant Blue G-250 binding assay (Bradford method; absorbance at 595 nm) as outlined by Bradford (1976). The proline accumulation was assessed through ninhydrin-acid reagent complexation (Bates protocol; toluene extraction at 520 nm) according to Bates et al. (1973). Superoxide dismutase (SOD) activity was measured spectrophotometrically at 560 nm using the nitroblue tetrazolium (NBT) photoreduction inhibition method (Giannopolitis and Ries, 1977), with enzyme activity units defined as the amount required to inhibit 50% of NBT reduction per minute (1 U = 50% inhibition/min). Peroxidase (POD) activity was measured spectrophotometrically at 470 nm through the guaiacol oxidation method (Omran, 1980), which quantifies enzymatic activity based on absorbance changes during the oxidation of guaiacol in the presence of hydrogen peroxide. Catalase (CAT) activity was measured spectrophotometrically at 240 nm using the hydrogen peroxide (H_2_O_2_) decomposition method (Aebi, 1984), which quantifies enzymatic activity based on the absorbance decrease caused by H_2_O_2_ degradation over time. Ascorbate peroxidase (APX) activity was determined spectrophotometrically at 290 nm using the ascorbate oxidation method, which quantifies enzymatic activity based on the absorbance decline resulting from the oxidation of ascorbate in the presence of hydrogen peroxide (H_2_O_2_).

#### 2.3.3 Malondialdehyde, superoxide anions, and hydrogen peroxide in leaves

Malondialdehyde (MDA) content was quantified according to Stewart and Bewley (1980) using the thiobarbituric acid (TBA) assay. Briefly, the homogenate was centrifuged to collect the supernatant, which was then mixed with 2 mL of 0.67% TBA. The mixture was heated at 100°C for 30 min, rapidly cooled in ice water, and centrifuged at 4000 rpm for 10 min. Absorbance of the resulting supernatant was measured at 532 nm (MDA-TBA adduct peak), 600 nm (turbidity correction), and 450 nm (non-specific interference correction). MDA concentration was calculated using an extinction coefficient of 155 mM^−1^ cm^−1^.

Superoxide anion (O_2_^−^) content was quantified using the hydroxylamine oxidation method (Wang and Jiao, 2000). Briefly, tissue samples were homogenized in 65 mM phosphate buffer (pH 7.8) and centrifuged at 12,000 ×g for 20 min. The supernatant was incubated with hydroxylamine hydrochloride (1 mM) in phosphate buffer for 40 min at 25°C, followed by sequential addition of p-aminobenzene sulfonic acid (3% w/v) and α-naphthylamine (0.2% w/v). The reaction mixture was shaken at 30°C for 30 min to facilitate diazo-coupling, after which an equal volume of chloroform was added to remove interfering pigments. Following centrifugation, and the absorbance was measured at 530 nm.

Hydrogen peroxide (H_2_O_2_) content was determined spectrophotometrically at 390 nm using the potassium iodide (KI) oxidation method (Velikova et al., 2000). Briefly, fresh tissue (0.5 g) was homogenized in 5% (w/v) trichloroacetic acid (TCA), centrifuged at 10,000 ×g for 15 min at 4°C, and the supernatant was mixed with 50 mM phosphate buffer (pH 7.0) and 1 M KI. The reaction mixture was incubated in darkness for 1 h. Absorbance was measured at 390 nm, with H_2_O_2_ concentration calculated using a standard curve.

#### 2.3.4 Measurement of nitrogen-metabolizing enzyme activities

The activity of nitrate reductase (NR), glutamine synthetase (GS), glutamate synthase (GOGAT), and glutamate dehydrogenase (GDH), were quantified at both the heading stage (HS) and filling stage (15 days post-heading, FS). The flag leaves per treatment were collected from rice plants, snap-frozen in liquid nitrogen, and stored at −80°C until analysis. Nitrate reductase (NR) activity was measured according to Hageman and Hucklesby (1971) by monitoring nitrite (NO_2_^−^) production at 540 nm and expressed as μmol NO_2_^−^·g^−1^ FW·h^−1^. Glutamine synthetase (GS) activity was determined via the hydroxamate synthetase method (Rowe et al., 1970), quantifying at 540 nm. Glutamate synthase (GOGAT) activity was assayed following Singh and Srivastava (1986) by tracking NADH oxidation at 340 nm. Glutamate dehydrogenase (GDH) activity was quantified using the NAD^+^-dependent amination reaction (Zou, 1995), measuring absorbance changes at 340 nm.

#### 2.3.5 Determination of nitrogen content

The plant samples of stem sheaths, leaves, and panicles collected at both heading and maturity stages were oven-dried and ground into powder. After digestion with H_2_SO_4_-H_2_O_2_, the nitrogen content in different organs and plants at various growth stages was determined using the semi-micro Kjeldahl method (Were et al., 2015).

#### 2.3.6 Measurement of rice biomass yield, grain yield, and yield attributes

At maturity stage in each experimental year, eight representative rice plants were randomly sampled from individual plots for biomass yield analysis. Following harvest, plant samples were subjected to a sequential dehydration process comprising 30 min at 105°C followed by equilibration at 60°C until constant mass attainment for biomass quantification. A 6 m² sampling area within each experimental plot was completely harvested to quantify rice grain yield. Grain yield was calculated based on total production per hectare using plot-scale conversion factors. Six intact panicles per plot were systematically selected for detailed yield attributes assessment following established protocols (Jin et al., 2018).

#### 2.3.7 Data calculation and statistical analysis

The total N content per plant was derived from the product of tissue N concentration and corresponding dry weight. Nitrogen transfer amount (leaves or stem sheath) after heading stage (kg hm^-2^) = nitrogen accumulation amount (leaves or stem sheath) at heading stage − nitrogen accumulation amount (leaves or stem sheath) at maturity stage; Nitrogen transport rate (%) = nitrogen transfer rate (leaves or stem sheath) ÷ Nitrogen accumulation at full heading stage (leaves or stem sheath) × 100. Nitrogen transport contribution rate (%) = (nitrogen transfer amount of leaf + nitrogen transfer amount of stem sheath) ÷ total nitrogen accumulation of panicle from heading stage to maturity stage × 100. Nitrogen use efficiency (NUE) = (N uptake in N application treatment- N uptake in blank area) ÷ nitrogen application amount × 100. Partial factor productivity of N fertilizer (PFPN, kg kg^-1^) = grain yield in fertilization area ÷ nitrogen applied amount. Nitrogen agronomic use efficiency (NAE, kg kg^-1^) = (grain yield in nitrogen application area − grain yield in nitrogen blank area) ÷ nitrogen application amount.

Statistical analyses were performed to evaluate treatment effects on the experimental parameters using one-way analysis of variance (ANOVA) followed by the least significant difference (LSD) testing. The potential interaction between treatment and temporal variations was examined through two-way ANOVA. All statistical procedures, including both ANOVA models and multiple comparison tests, were implemented using SPSS 18.0 statistical software (IBM Corp., Armonk, NY, USA) following standard protocols for agricultural experimental design. Pearson correlation analysis, principal component analysis, and the generation of plot figures were performed using Origin Pro 2023 (OriginLab. Inc., USA).

## 3 Results

### 3.1 Ionic balance

**Figure 1:**
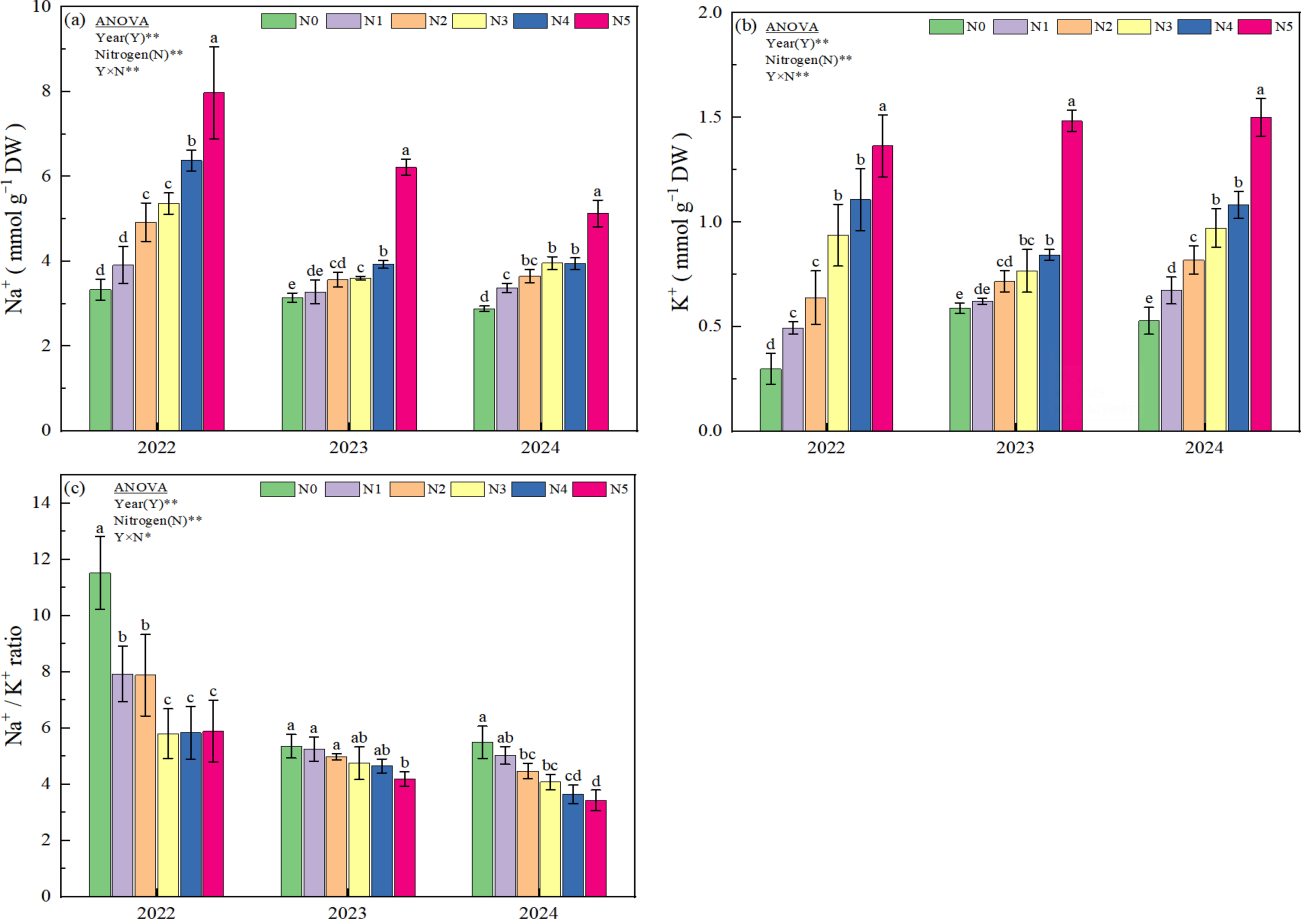
Effect of different nitrogen application amounts on Na^+^ concentration **(a)**, K^+^ concentration **(b)**, and Na^+^/K^+^ ratio **(c)**. N0, N1, N2, N3, N4, and N5 represent 0, 125, 175, 225, 275, and 325 kg ha^-1^ of N, respectively. ** denotes significant at the 0.01 level, and * denotes significant at the 0.05 level. Lowercase letters **(a-e)** above the error line indicate differences between N fertilizer treatments (p<0.05).

N fertilizer application significantly increased Na^+^ and K^+^ concentrations and significantly decreased Na^+^/K^+^ ratio in rice leaves in 2022-2024 (**Figure 1**). The Na^+^ and K^+^ concentrations exhibited a consistent ascending order across nitrogen treatments: N0 < N1 < N2 < N3 < N4 < N5 (**Figures 1a, b**). ANOVA analysis revealed that N5 demonstrated statistically significant differences (p<0.05) other treatments (N0-N4) in both ionic species. Intriguingly, nitrogen supplementation paradoxically reduced the Na^+^/K^+^ ratio compared to N0. In 2022, the Na^+^/K^+^ ratio decreased by 31.16% (N1), 31.51% (N2), 49.57% (N3), 49.31% (N4), and 48.87% (N5). This suppression pattern persisted in subsequent growing seasons, with reductions of 1.87-21.64% (2023) and 8.55-37.64% (2024) observed across treatments (**Figure 1c**). Additionally, two-way ANOVA revealed significant interactive effects (p<0.05) between nitrogen fertilization amount and year on sodium (Na^+^) and potassium (K^+^) concentrations, as well as Na^+^/K^+^ ratio.

### 3.2 The concentration of malondialdehyde, superoxide anions, and hydrogen peroxide

**Figure 2:**
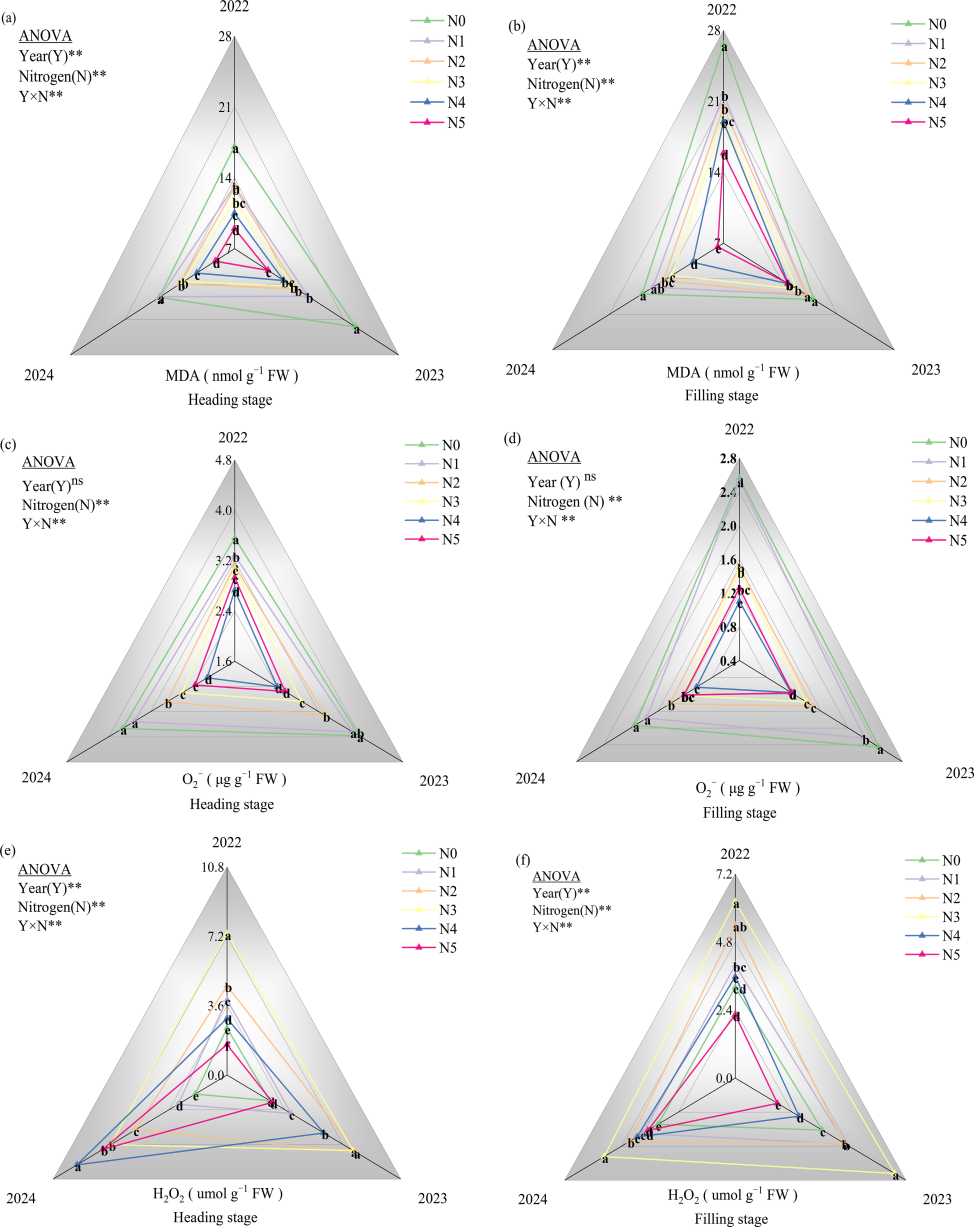
Effect of N fertilizer on MDA **(a, b)**, O_2_^-^ **(c, d)**, and H_2_O_2_ **(e, f)** at heading and filling stage. N0, N1, N2, N3, N4, and N5 represent 0, 125, 175, 225, 275, and 325 kg ha-1 of N, respectively. ** denotes significant at the 0.01 level, and ns denotes not significant. Lowercase letters (a-e) above the error line indicate differences between N fertilizer treatments (p<0.05).

Nitrogen application significantly reduced MDA concentration at both heading and filling stages (**Figures 2a, b**). Compared to the non-fertilized control (N0), the N1-N5 treatments induced progressive reductions of 17.98%, 25.35%, 29.51%, 36.61%, and 47.07% at heading stage (**Figure 2a**), with corresponding decreases of 11.57%, 15.80%, 21.34%, 27.47%, and 37.63% observed at filling stage (**Figure 2b**). Notably, this suppressive effect reached statistical significance (p<0.05) starting from the moderate nitrogen level (N3), with more pronounced differences in high nitrogen applied amount treatments (N4-N5) compared to the N0. The concentration of O_2_^-^ showed a decreased trend with increased N application, and followed the order of N0 > N1 > N2 > N3 > N5 > N4 (**Figures 2c, d**). At heading stage, N1-N5 treatments reduced O_2_^−^ levels by 5.59% to 35.64% respectively compared to the N0 (**Figure 2c**), while filling stage exhibited more pronounced reductions ranging from 6.04% to 52.59% (**Figure 2d**). The effects of different N applied treatments on H_2_O_2_ concentration were not consistent under different experimental year (**Figures 2e, f**). In 2022, H_2_O_2_ accumulation showed an increasing trend with the increase of nitrogen application amount, peaking at N3 before declining at the highest application rate (N5). At the heading stage, all nitrogen treatments demonstrated statistically significant differences in H_2_O_2_ relative to N0 (p<0.05) (**Figure 2e**). Contrastingly, at filling stage, significant variations (p < 0.05) were specifically observed in N2 and N3 treatments compared to N0 (**Figure 2f**). In 2023, H_2_O_2_ concentration at the heading stage displayed a hierarchical response to nitrogen treatments (N2 > N3 > N4 > N1 > N5 > N0). Treatments N1-N4 demonstrated statistically significant differences (p < 0.05) compared to the control (N0), while N5 showed no significant variation. In 2024, H_2_O_2_ concentration exhibited distinct patterns across nitrogen treatments. At the heading stage, the H_2_O_2_concentrations significantly increased from N0 to N4, reaching maximum levels at N4 (p<0.05). Conversely, at filling stage, peak concentrations occurred at N3 treatment. Statistical analysis revealed that all nitrogen application groups (N1-N5) showed significantly elevated H_2_O_2_ levels compared to the N0, with differentials reaching statistical significance (p<0.05). A significant interactive effect of nitrogen (N) and year (Y) on MDA, O_2_^-^, and H_2_O_2_ were observed.

### 3.3 The content of soluble sugar, soluble protein, and proline

**Figure 3:**
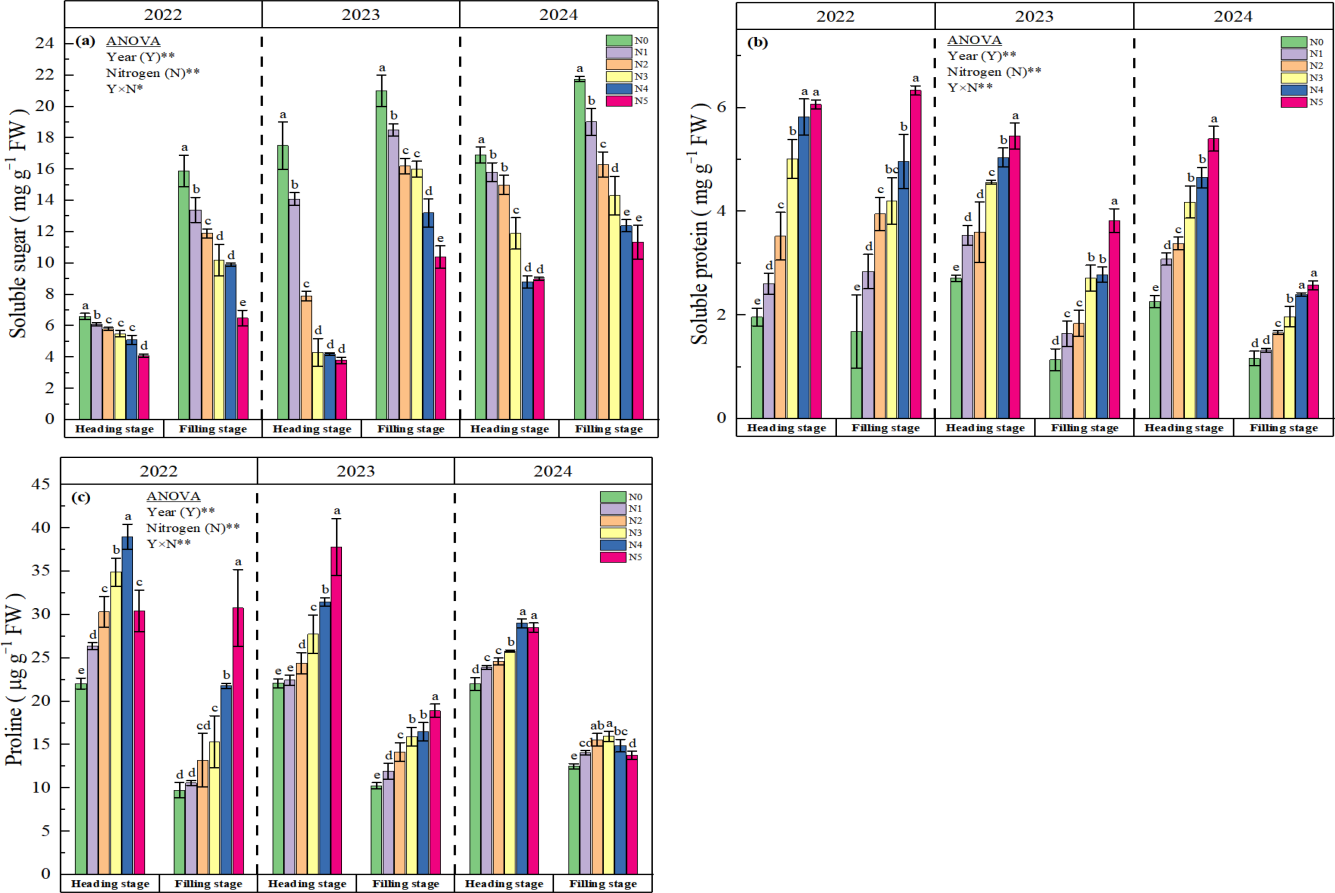
Effect of N fertilizer on soluble sugar **(a)**, soluble protein **(b)**, and proline **(c)**. N0, N1, N2, N3, N4, and N5 represent 0, 125, 175, 225, 275, and 325 kg ha^-1^ of N, respectively. ** denotes significant at the 0.01 level, and ns denotes not significant. Lowercase letters **(a-e)** above the error line indicate differences between N fertilizer treatments (p<0.05).

The soluble sugar content in rice cultivated under soda saline-alkali paddy soils exhibited a significant decreasing trend with incremental nitrogen application amounts (**Figure 3a**). Comparative analysis revealed that nitrogen treatments (N1-N5) induced reductions of 12.22%, 29.99%, 47.11%, 55.89%, and 58.81% at the heading stage, respectively, compared to the control (N0). During the filling stage, the reduction range expanded to 13.20-51.87% across treatments. All nitrogen-applied treatments (N1-N5) showed statistically significant differences (p<0.05) in soluble sugar content relative to the non-nitrogen treatment throughout both growth stages. The soluble protein content demonstrated a progressive elevation with increasing nitrogen application levels (**Figure 3b**), and exhibiting a trend of N5 > N4 > N3 > N2 > N1 > N0. Specifically, nitrogen treatments (N1-N5) enhanced soluble protein content by 32.90%, 51.52%, 98.27%, 123.81%, and 144.16% at the heading stage, and by 45.11%, 86.47%, 122.56%, 154.14%, and 218.80% at the filling stage, respectively (three-year mean values). All nitrogen-applied treatments exhibited statistically significant differences (p<0.05) compared to the N0 at heading stage. The application of nitrogen fertilizer significantly modulated leaf proline content, with notable interannual variability observed across the three-year study period (2022-2024) (**Figure 3c**). During the 2022 filling stage and both growth stages in 2023, proline content exhibited a consistent ascending pattern from N0 to N5. Specifically, N3-N5 demonstrated statistically significant elevations (p<0.05), compared to N0. Contrastingly, at heading stage in 2022 and 2024, proline content reached maximum values at N4 before declining at N5. At filling stage in 2024, showed an order of N3 > N2 > N4 > N1 > N5 > N0. Two-way ANOVA analysis showed that nitrogen (N) and year (Y) had significant interaction effects on soluble sugar, soluble protein and proline content (p<0.05).

### 3.4 The activity of superoxide dismutase, peroxidase, catalase, and ascorbate peroxidase

**Figure 4:**
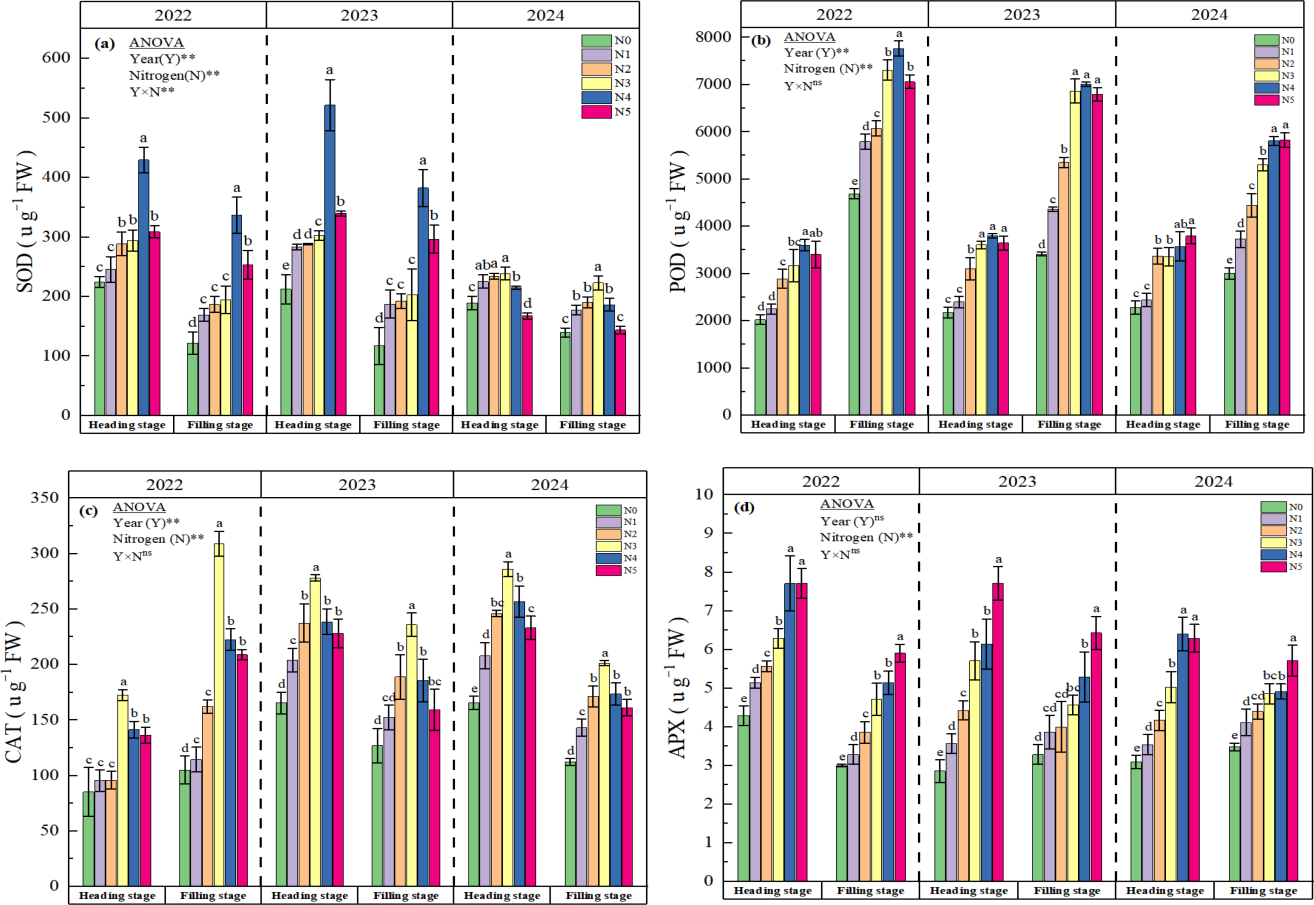
Effect of N fertilizer on SOD **(a)**, POD **(b)**, CAT **(c)** and APX **(d)**. N0, N1, N2, N3, N4, and N5 represent 0, 125, 175, 225, 275, and 325 kg ha^-1^ of N, respectively. ** denotes significant at the 0.01 level, and ns denotes not significant. Lowercase letters (a-e) above the error line indicate differences between N fertilizer treatments (p<0.05).

The SOD exhibited a distinct ranking across nitrogen applied treatments: N4 > N5 > N3 > N2 > N1 > N0 in 2022 and 2023. Treatments N1-N5 demonstrated statistically significant differences (p < 0.05) compared to theN0 (**Figure 4a**). However, with the increase of planting years (in 2024), SOD in rice showed a significant decline trend, especially in N5 treatment. The results of the two-way ANOVA underscored the SOD significant influence of nitrogen, year, and the interaction between nitrogen treatment and year on SOD. The average POD in N1, N2, N3, N4 and N5 were increased by 9.36-69.42% at heading stage, and by 24.95-85.21% at filling stage, respectively. The difference between N2-N5 and N0 reached a significant level from 2022 to 2024 (p<0.05). POD activity demonstrated significant temporal variation across experimental years at critical rice growth stages. Compared to the 2022, heading-stage POD increased by 7.97% (2023) and 8.52% (2024), whereas filling-stage activity exhibited contrasting patterns with reductions of 12.64% (2023) and 27.29% (2024) (**Figure 4b**). No significant interactions between nitrogen treatment and year on POD. In three consecutive years of this experiment, with the increase of nitrogen application amount, CAT showed a significant increase trend at heading and filling stage (**Figure 4c**), especially N3 treatment was significantly higher than other treatments (p<0.05). Moreover, CAT exhibited pronounced interannual variation. At heading-stage, CAT showed progressive annual enhancement, whereas filling-stage displayed significant interannual depletion. Compared to N0, the average APX in N1, N2, N3, N4 and N5 were increased by 19.65%, 38.42%, 66.28%, 97.95% and 112.32% at heading stage, and by 15.03%, 25.46%, 44.48%, 56.75% and 84.66% at filling stage, respectively (**Figure 4d**). The difference between nitrogen application treatment (N1-N5) and N0 reached a significant level at both growth stages in all experimental years. Furthermore, with the increase of planting years, APX showed a significant downward trend at heading stage, whereas filling stage exhibited a significant upward trend.

### 3.5 The activity of nitrogen-metabolizing enzyme

**Figure 5:**
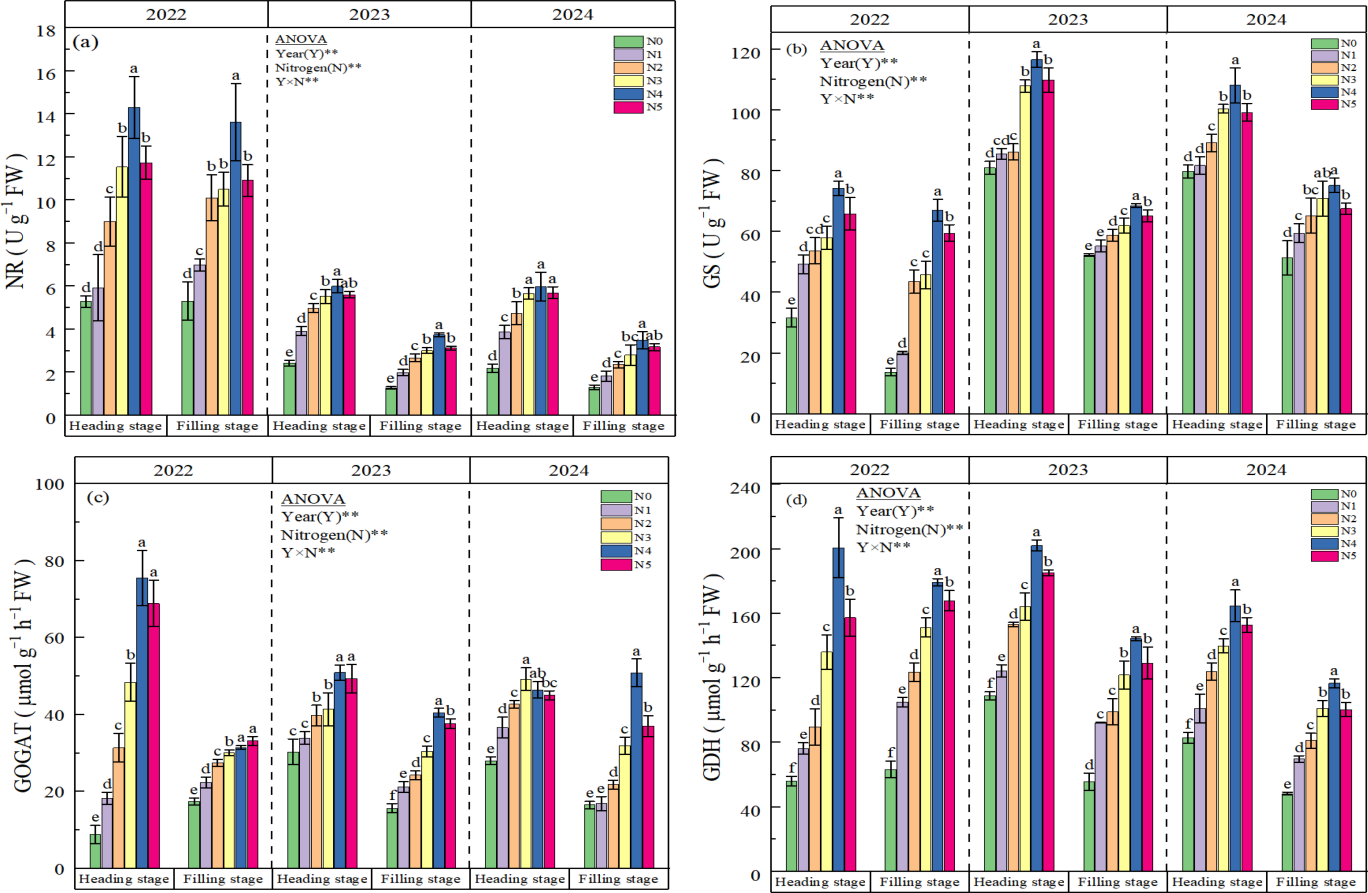
Effect of N fertilizer on NR **(a)**, GS **(b)**, GOGAT **(c)** and GDH **(d)**. N0, N1, N2, N3, N4, and N5 represent 0, 125, 175, 225, 275, and 325 kg ha^-1^ of N, respectively. ** denotes significant at the 0.01 level, and ns denotes not significant. Lowercase letters (a-f) above the error line indicate differences between N fertilizer treatments (p<0.05).

NR activity demonstrated dose-responsive enhancement under nitrogen fertilization. At heading stage, N1-N5 treatments showed increases of 38.37%, 88.52%, 128.70%, 164.65%, and 132.02% respectively compared to N0, while it exhibited 36.88%-118.25% elevation across treatments at filling stage (**Figure 5a**). Statistical analysis confirmed significant treatment effects (N1-N5 vs. N0, p<0.05) across both growth stages, with the notable exception of 2022 heading stage observations where N1 failed to achieve significance. Moreover, with the increase of planting years, the NR activity of rice at both growth stages showed a significant decline trend. At both growth stages, the GS activity exhibiting a trend of N4 > N5 > N3 > N2 > N1 > N0. However, in 2024, N3 exhibited higher GS activity than N5 (**Figure 5b**). In 2022, all nitrogen treatments (N1, N2, N3, N4, N5) showed statistically significant differences (p<0.05) compared to N0. By contrast, in 2023 and 2024, the difference between N1 and N0 was no longer statistically significant. Moreover, with the increase of planting years, GS in rice showed a significant increase trend. Compared with 2022, GS increased by 76.36% (2023) and 67.78% (2024) at heading, and by 44.93% (2023) and 54.46% (2024) at filling stage, respectively (**Figure 5b**). Nitrogen treatments did not show consistent effects on GOGAT activity in rice leaves during heading and filling stages from 2022 to 2024 (**Figure 5c**). The trend of GOGAT activity followed N4 > N5 > N3 > N2 > N1 > N0 at the heading stage in 2022, both heading and filling stages in 2023, and the filling stage in 2024. Significant differences (p<0.05) were observed between N2, N3, N4, N5 treatments and N0 in these stages. However, at the filling stage in 2022, the N5 treatment showed higher GOGAT activity than N4, though this difference was not statistically significant. In contrast, at the filling stage in 2024, GOGAT activity peaked in the N3 treatment and subsequently decreased in N4, with all N treatments showing significant differences (p<0.05) compared to N0. Compared to N0, the average activity of GDH in the N1, N2, N3, N4, and N5 treatments increased by 21.65%, 47.90%, 77.51%, 128.81%, and 99.79% at the heading stage, and by 59.66%, 81.69%, 123.82%, 163.41%, and 135.43% at the filling stage, respectively (**Figure 5d**). Compared with 2022, GDH activity increased by 31.02% (2023) and 6.91% (2024) at heading stage; however, it decreased by 18.75% (2023) and 35.04% (2024) at filling stage, respectively. A significant interactive effect of nitrogen (N) and year (Y) on NR, GS, GOGAT, and GDH were observed.

### 3.6 N translocation of rice from heading stage to mature stage

The average of N translocation amount in stems and leaves initially increased from the N0 (no fertilizer) to N4 treatments, peaking at N4, before declining at the N5 level. Compared to N0, the N translocation amounts in stems under N1-N5 treatments increased by 98.10%, 147.87%, 196.45%, 245.26%, and 159.24%, respectively. Similarly, leaf N translocation amounts rose by 71.60%, 137.20%, 210.80%, 345.20%, and 232.40% for N1-N5, respectively, compared to N0 (**Table 2**). The N translocation amount of stems and leaves treated with N fertilization (N1-N5) was significantly different from that treated without N fertilization (N0). Compared to 2022, N translocation amount of stems and leaves increased by 25.86% (2023) and 103.26% (2024), and by 156.91% (2023) and 151.73% (2024), respectively (**Table 2**). The N translocation efficiency exhibited inconsistency across different planting years and organs. In 2022, the N translocation efficiency of stems and leaves exhibited an increasing trend across nitrogen treatments, with the N4 treatment demonstrating the highest performance. In contrast, in 2023, the stem N translocation efficiency followed the order N0 > N1 > N2 > N3 > N4 > N5, with significant differences observed between N3-N5 and the N0 treatment (p<0.05). The N translocation efficiency in leaves increased with nitrogen application rate, although no significant differences were observed among treatments. The N translocation efficiency in stems showed a significant decreasing trend with increasing planting years. With increasing N application rates, the average contribution of N translocation to panicle initially increased then decreased. Compared to the N0 control, the N1-N5 treatments showed increases of 15.01%, 28.82%, 25.29%, 21.51%, and 6.34%, respectively. Two-way ANOVA revealed significant effects of nitrogen (N), year (Y), and their interaction (N×Y) on N translocation amount, N translocation efficiency, and contribution of N translocation to panicle.

**Table 2:** Effects of N fertilizer on N translocation of rice from heading stage to mature stage in soda saline-alkali paddy fields.

Year	N applied level	Heading stage to mature stage				Contribution of N translocation to the panicle (%)
		Stems		Leaves		
		N translocation amount (kg ha^-1^)	N translocation efficiency (%)	N translocation amount (kg hm^-2^)	N translocation efficiency (%)	
2022	N0	0.97 ± 0.32 d	63.43 ± 3.05 b	0.62 ± 0.01 e	62.74 ± 4.96 c	39.57 ± 1.12 c
	N1	3.95 ± 0.13 c	65.26 ± 4.63 ab	1.23 ± 0.22 d	65.17 ± 1.40 bc	43.46 ± 4.11 bc
	N2	5.41 ± 0.14 c	68.69 ± 2.34 a	2.64 ± 1.02 c	66.60 ± 3.04 bc	53.15 ± 1.98 a
	N3	7.96 ± 0.59 b	69.98 ± 5.20 a	3.59 ± 0.29 bc	68.51 ± 1.01 b	53.88 ± 3.07 a
	N4	14.69 ± 1.75 a	70.49 ± 1.02 a	7.27 ± 1.06 a	73.85 ± 3.02 a	56.43 ± 2.18 a
	N5	9.71 ± 2.14 b	65.69 ± 6.41 ab	4.33 ± 0.34 b	68.12 ± 1.02 b	49.06 ± 10.14 ab
2023	N0	5.98 ± 0.28 c	58.67 ± 1.52 a	3.46 ± 0.25 e	66.20 ± 4.82 a	40.68 ± 0.62 a
	N1	8.67 ± 1.50 ab	55.16 ± 2.92 a	5.66 ± 0.96 d	67.92 ± 5.39 a	42.89 ± 1.08 a
	N2	10.43 ± 1.27 ab	53.13 ± 4.65 ab	6.98 ± 0.23 c	66.86 ± 4.91 a	42.62 ± 2.95 a
	N3	10.76 ± 1.27 a	46.98 ± 1.92 b	10.76 ± 0.53 b	72.14 ± 3.37 a	42.83 ± 1.82 a
	N4	10.10 ± 1.48 ab	35.10 ± 4.37 c	12.48 ± 0.86 a	69.19 ± 1.38 a	33.78 ± 2.67 b
	N5	7.79 ± 2.15 bc	31.23 ± 7.61 c	11.22 ± 0.80 b	70.06 ± 3.32 a	32.93 ± 5.10 b
2024	N0	5.72 ± 0.58 d	58.92 ± 1.71 b	3.41 ± 0.16 d	69.59 ± 1.72 bc	41.44 ± 3.84 d
	N1	12.45 ± 0.20 c	71.36 ± 2.56 a	5.99 ± 0.47 c	73.62 ± 1.17 ab	53.61 ± 3.17 bc
	N2	15.53 ± 1.88 b	71.42 ± 2.73 a	8.16 ± 0.16 b	74.71 ± 0.79 a	60.99 ± 6.40 a
	N3	18.82 ± 0.95 a	68.29 ± 2.83 a	8.95 ± 1.13 b	68.48 ± 3.36 c	55.75 ± 3.80 ab
	N4	18.93 ± 1.06 a	59.22 ± 2.06 b	13.65 ± 1.31 a	73.65 ± 1.48 ab	57.65 ± 0.72 ab
	N5	15.32 ± 0.89 b	55.83 ± 3.77 b	9.38 ± 0.97 b	67.04 ± 3.88 c	47.42 ± 2.86 cd
ANOVA						
Year (Y)		**	**	**	**	**
Nitrogen (N)		**	**	**	**	**
Y×N		**	**	**	**	**

N0, N1, N2, N3, N4, and N5 represent 0, 125, 175, 225, 275, and 325 kg ha^-1^ of N, respectively.

Lower case letters indicate significant differences between different N application treatments within the same year (P = 0.05). **, significant the 0.01 probability level, respectively.

### 3.7 Nitrogen uptake and utilization efficiency of rice

Increased nitrogen application amount significantly enhanced total N uptake in rice grown in soda saline-alkali paddy soils (**Table 3**). Throughout the three-year experiment (2022-2024), total N uptake of rice consistently followed the order: N4 > N5 > N3 > N2 > N1 > N0, with statistically significant differences observed among all treatments (p<0.05). Furthermore, total N uptake of rice showed a significant increasing trend with successive years of rice planting. Field experiments conducted from 2022 to 2024 revealed that nitrogen use efficiency (NUE) consistently followed the order: N4 > N3 > N5 > N2 > N1. Significant differences (p<0.05) were observed between N3-N5 and N1-N2 treatments. Notably, in 2022 and 2023, the N4 treatment showed significantly higher NUE than both N3 and N5 treatments. Compared with 2022, NUE increased by 45.10% in 2023 and 31.80% in 2024. In both 2022 and 2023, nitrogen agronomic efficiency (NAE) followed the order N4 > N3 > N2 > N1 > N5, while a similar trend (N4 > N3 > N2 > N1 > N5) was observed in 2024. Throughout the three-year study, N4 treatment showed significantly higher NAE compared to N2, N1, and N5 treatments (p<0.05). Interannual comparisons revealed that NAE in 2023 was significantly increased relative to 2022, whereas a decreasing trend emerged in 2024. During the three-year field experiment, PEPN decreased with increasing nitrogen application rates. Compared to N1, the average PFPN values for N2, N3, N4, and N5 were reduced by 20.61%, 30.12%, 28.77%, and 49.65%, respectively. Significant differences (p<0.05) were observed between N2-N5 and N1 treatments, with N5 showing significantly lower than N2, N3, and N4 (p<0.05). A significant interactive effect of nitrogen (N) and year (Y) on total N uptake and NUE were observed.

**Table 3:** Effect of N fertilizer on nitrogen uptake and use efficiency of rice in soda saline-alkali paddy fields in 2022-2024.

Year	N applied level	Total N uptake (kg ha^-1^)	NUE (%)	NAE (kg kg^-1^)	PFPN (kg kg^-1^)
2022	N0	5.34 ± 0.56 f			
	N1	15.98 ± 1.18 e	8.51 ± 0.52 c	7.20 ± 0.80 bc	36.27 ± 3.61 a
	N2	20.49 ± 1.16 d	8.65 ± 0.38 c	7.24 ± 0.33 bc	28.00 ± 2.62 b
	N3	30.04 ± 1.97 c	10.98 ± 1.08 b	8.00 ± 0.77 b	24.15 ± 1.43 b
	N4	53.07 ± 2.08 a	17.35 ± 0.60 a	11.27 ± 1.45 a	24.49 ± 1.83 b
	N5	39.23 ± 0.54 b	10.43 ± 0.16 b	6.26 ± 0.36 c	17.44 ± 0.94 c
2023	N0	28.94 ± 1.37 f			
	N1	43.04 ± 1.10 e	12.19 ± 0.61 d	7.73 ± 1.22 c	31.73 ± 0.46 a
	N2	53.48 ± 1.06 d	14.03 ± 1.22 cd	8.57 ± 1.51 bc	25.72 ± 0.99 b
	N3	66.55 ± 3.05 c	16.72 ± 1.48 b	10.67 ± 2.67 ab	24.00 ± 1.78 bc
	N4	91.08 ± 1.78 a	22.60 ± 0.16 a	12.61 ± 0.21 a	23.52 ± 0.56 c
	N5	79.63 ± 2.77 b	15.60 ± 1.21 bc	7.59 ± 0.94 c	16.82 ± 0.36 d
2024	N0	30.12 ± 0.87 f			
	N1	44.39 ± 2.18 e	11.41 ± 1.12 c	5.59 ± 1.15 b	31.98 ± 1.23 a
	N2	52.80 ± 0.94 d	12.96 ± 0.99 bc	6.80 ± 0.79 b	25.66 ± 1.00 b
	N3	69.50 ± 1.69 c	17.50 ± 0.73 a	7.06 ± 0.63 b	23.73 ± 0.72 c
	N4	80.69 ± 1.86 a	18.39 ± 0.39 a	11.23 ± 0.69 a	23.22 ± 0.54 c
	N5	73.82 ± 3.39 b	13.44 ± 1.09 b	5.93 ± 0.46 b	16.08 ± 0.34 d
ANOVA					
Year (Y)		**	**	**	**
Nitrogen (N)		**	**	**	**
Y×N		**	**	ns	ns

N0, N1, N2, N3, N4, and N5 represent 0, 125, 175, 225, 275, and 325 kg ha^-1^ of N, respectively.

NUE: nitrogen use efficiency, NAE: nitrogen agronomic use efficiency, PFPN: partial factor productivity of N fertilizer.

Lower case letters indicate significant differences between different N application treatments within the same year (P = 0.05).

**, significant at the 0.01 probability level, respectively, ns, no significant at the 0.05 probability level.

### 3.8 Grain yield and its component factors

Applying nitrogen fertilizer treatments (N1-N5) significantly increased the number of panicles and grains per panicle in rice cultivated on soda saline-alkali paddy soils (**Table 4**). Compared with the N0, the increases in number of panicles for N5, N4, N3, N2, and N1 were 43.80%, 62.48%, 41.83%, 34.96%, and 32.26%, respectively. Notably, the N4 treatment showed a statistically significant difference compared to all other treatments (P<0.05). Furthermore, there was a significant interaction effect between nitrogen application and the planting year on the number of panicles. Compared to 2022, the number of panicles in 2023 showed a slight decreasing trend, whereas an increasing trend was observed in 2024. Relative to N0, the number of grains per panicle in treatments N5, N4, N3, N2, and N1 increased by 55.85% to 24.63%. Notably, the N4 and N5 treatments exhibited statistically significant differences compared to the N2 and N1 treatments. The seed setting rate exhibited inconsistencies across different planting years. In the first year of cultivation (2022), the N0 treatment had the lowest rate, with significant differences observed between nitrogen fertilizer treatments (N1-N5) and the no nitrogen application treatment (N0). In the second year (2023), the N5 treatment showed the lowest seed setting rate, while in the third year (2024), no significant differences were found among the various treatments. Additionally, the planting years did not have a significant effect on the seed setting rate of rice grown in soda saline-alkali soils. Neither nitrogen application treatments nor planting years had a significant effect on the 1000-grain weight of rice. Nitrogen application treatments and planting years had significant effects on rice grain yield in soda saline-alkali soil (p<0.05). Over the three-year trial period (2022–2024), the grain yield followed the order N4 > N5 > N3 > N2 > N1 > N0. Compared to the N0 treatment, grain yields under N4, N5, N3, N2, and N1 increased by 94.34%, 62.40%, 56.15%, 37.78%, and 24.01%, respectively. Significant differences were observed between all nitrogen treatments (N1–N5) and the no-nitrogen treatment (N0), with the N4 treatment differing significantly from all other treatments. Two-way ANOVA indicated no significant interaction between nitrogen fertilizer application and planting year on rice grain yield.

**Table 4:** Effect of N fertilizer on yield and components of rice in soda saline-alkali paddy fields.

Year	N applied level	Number of panicles (10^4^ ha^-1^)	Number of grains per panicle	Seed setting rate (%)	1000-grain weight (g)	Grain yield (t ha^-1^)
2022	N0	119.67 ± 8.39 b	68.47 ± 0.81 c	87.68 ± 4.71 b	28.14 ± 2.81 a	3.63 ± 0.42 e
	N1	219.80 ± 12.73 a	84.80 ± 4.47 b	92.84 ± 0.80 a	28.31 ± 0.63 a	4.53 ± 0.15 d
	N2	221.67 ± 20.00 a	93.80 ± 3.91 b	92.20 ± 1.27 a	27.15 ± 0.55 a	4.90 ± 0.46 cd
	N3	236.33 ± 1.28 a	99.27 ± 4.12 ab	92.64 ± 0.82 a	27.91 ± 2.65 a	5.43 ± 0.32 bc
	N4	244.17 ± 15.43 a	105.13 ± 6.18 a	92.75 ± 1.55 a	27.17 ± 0.64 a	6.73 ± 0.50 a
	N5	238.00 ± 3.61 a	99.07 ± 4.12 ab	93.74 ± 1.67 a	25.70 ± 2.42 a	5.67 ± 0.31 b
2023	N0	149.33 ± 3.61 d	58.13 ± 1.36 d	92.90 ± 0.93 ab	26.31 ± 0.57 a	3.00 ± 0.20 e
	N1	170.65 ± 6.36 c	75.40 ± 2.25 c	95.36 ± 1.32 a	26.83 ± 0.62 a	3.97 ± 0.06 d
	N2	172.80 ± 5.52 c	83.93 ± 1.10 b	93.52 ± 0.67 ab	26.12 ± 1.55 a	4.50 ± 0.17 c
	N3	188.90 ± 8.81 b	84.47 ± 2.53 b	91.49 ± 0.69 b	25.62 ± 1.24 a	5.42 ± 0.31 b
	N4	232.97 ± 8.08 a	93.00 ± 4.66 a	93.81 ± 1.95 ab	25.38 ± 1.26 a	6.47 ± 0.15 a
	N5	194.60 ± 5.90 b	85.27 ± 4.16 ab	86.77 ± 2.09 c	25.53 ± 0.43 a	5.47 ± 0.12 b
2024	N0	189.00 ± 3.61 c	59.47 ± 9.47 e	86.02 ± 1.33 a	23.47 ± 3.44 a	3.45 ± 0.25 f
	N1	215.33 ± 5.51 b	71.70 ± 4.16 d	86.20 ± 0.66 a	23.97 ± 0.21 a	4.00 ± 0.15 e
	N2	223.67 ± 6.66 b	76.17 ± 2.01 cd	87.06 ± 1.22 a	25.23 ± 1.68 a	4.49 ± 0.18 d
	N3	224.33 ± 10.21 b	81.73 ± 1.50 bc	86.84 ± 1.46 a	23.64 ± 1.12 a	4.89 ± 0.16 c
	N4	267.00 ± 11.67 a	91.87 ± 1.46 a	84.76 ± 0.62 a	25.60 ± 1.09 a	6.39 ± 0.15 a
	N5	226.00 ± 22.61 b	86.47 ± 1.00 ab	83.20 ± 2.55 a	25.86 ± 2.28 a	5.23 ± 0.11 b
ANOVA						
Year (Y)		*	*	ns	ns	*
Nitrogen (N)		**	**	*	ns	**
Y×N		**	ns	ns	ns	ns

N0, N1, N2, N3, N4, and N5 represent 0, 125, 175, 225, 275, and 325 kg ha^-1^ of N, respectively.

Lower case letters indicate significant differences between different N application treatments within the same year (P = 0.05).

* and **, significant at the 0.05 and 0.01 probability level, respectively. ns, no significant at the 0.05 probability level.

### 3.9 Correlations among ion concentrations, physiological parameter, nitrogen use efficiency, and grain yield

**Figure 6:**
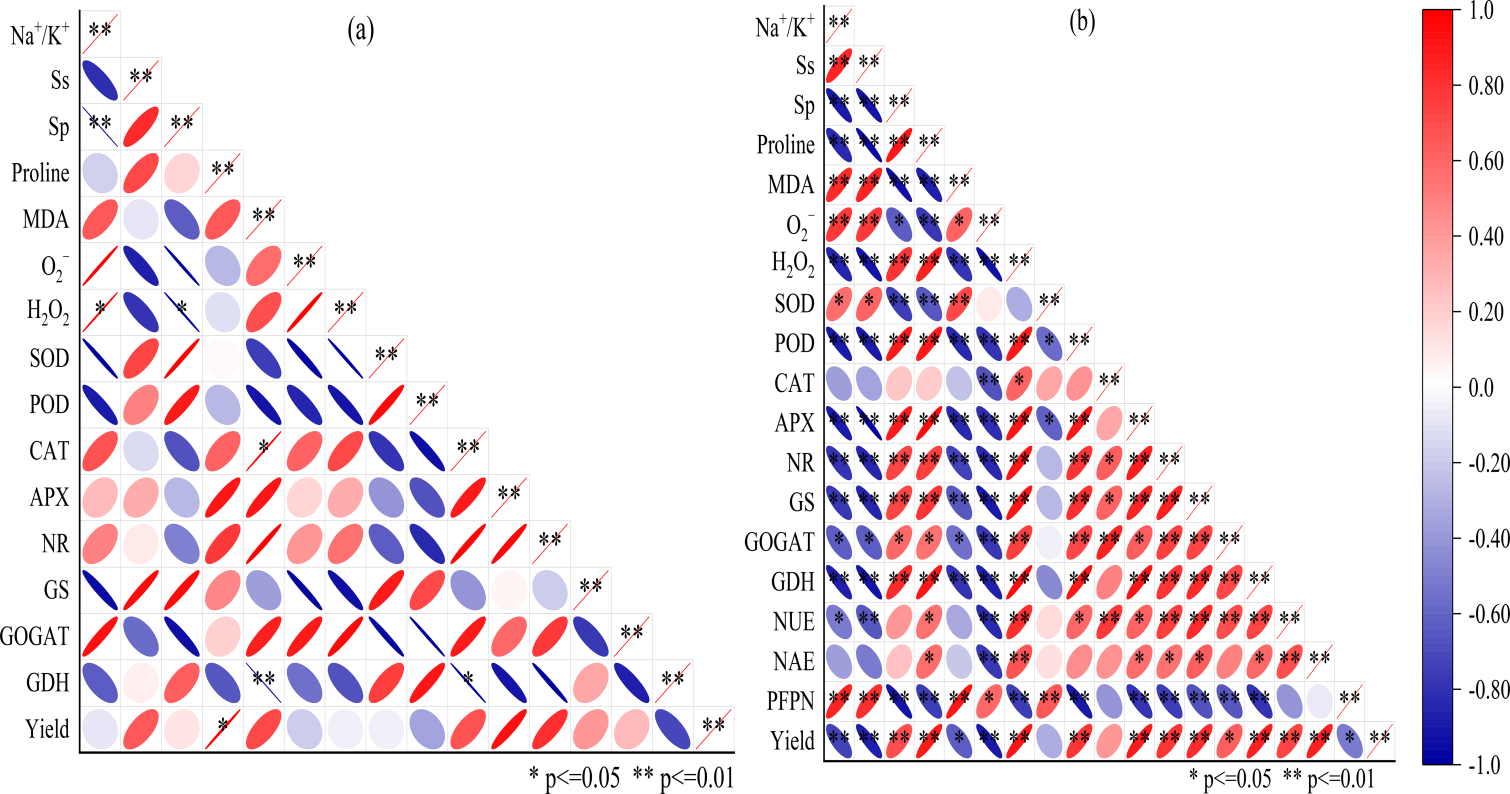
Pearson correlation analysis among yield, nitrogen use efficiency, Na^+^/K^+^ ratio, physiological parameter, and grain yield. **(a)** indicated to non-nitrogen fertilizer treatment, **(b)** indicated to the treatments with nitrogen application. *, ** Correlation is significant at the P < 0.05 and 0.01 level, respectively. Ss, soluble sugar; Sp, soluble protein; MDA, malondialdehyde; O_2_^−^, superoxide anions; H_2_O_2_, hydrogen peroxide; SOD, superoxide dismutase; POD, peroxidase; CAT, catalase; APX, ascorbate peroxidase; NR, nitrate reductase; GS, glutamine synthetase; GOGAT, glutamate synthase; GDH, glutamate dehydrogenase; NUE, nitrogen use efficiency; NAE, nitrogen agronomic use efficiency; PFPN, partial factor productivity of N fertilizer.

**Figure 7:**
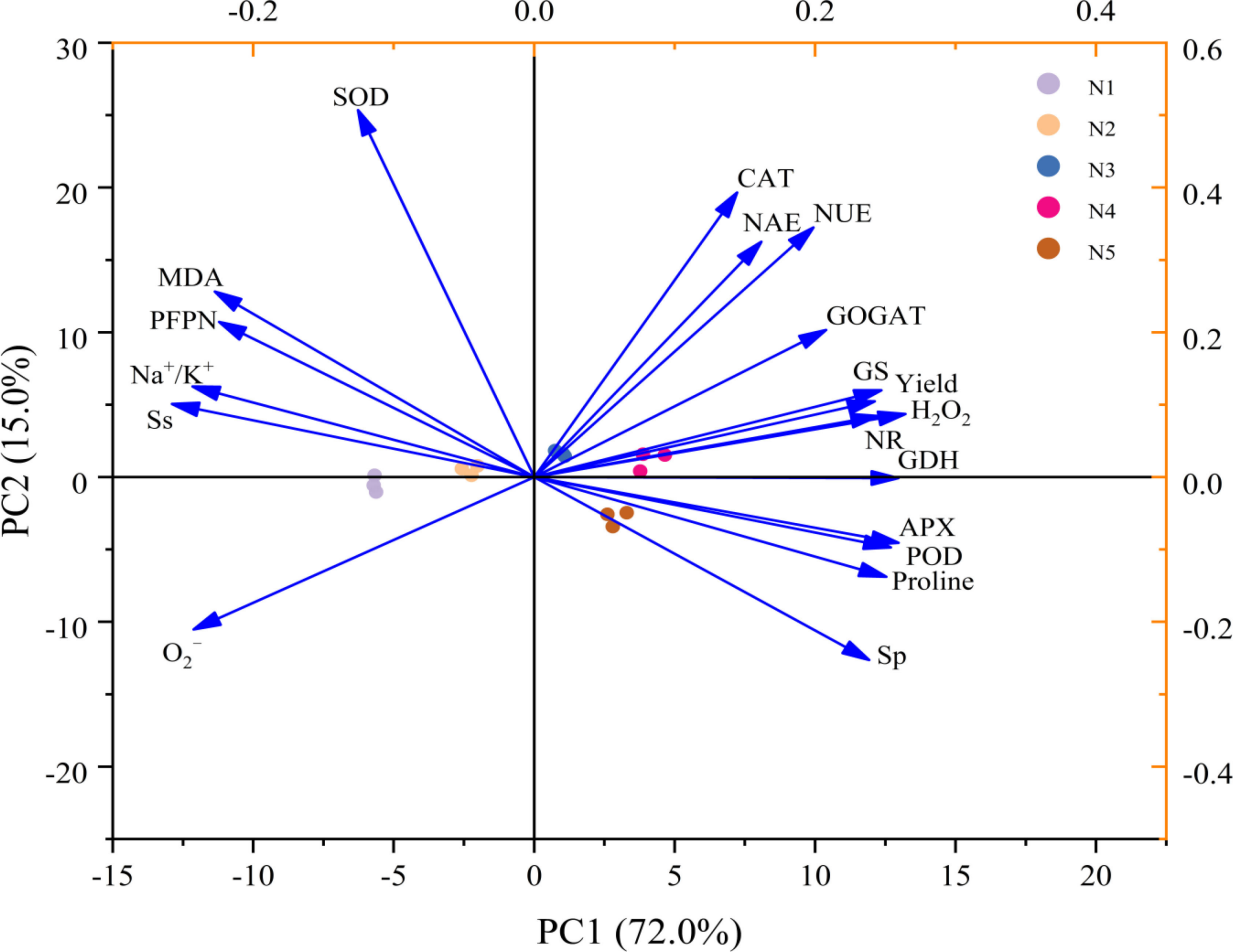
Principal component analysis of yield, nitrogen use efficiency, Na^+^/K^+^ ratio, physiological parameter, and grain yield. Ss, soluble sugar; Sp, soluble protein; MDA, malondialdehyde; O_2_^−^, superoxide anions; H_2_O_2_, hydrogen peroxide; SOD, superoxide dismutase; POD, peroxidase; CAT, catalase; APX, ascorbate peroxidase; NR, nitrate reductase; GS, glutamine synthetase; GOGAT, glutamate synthase; GDH, glutamate dehydrogenase; NUE, nitrogen use efficiency; NAE, nitrogen agronomic use efficiency; PFPN, partial factor productivity of N fertilizer.

**Figure 6** illustrates that yield exhibited positive correlations with SP, Proline, CAT, NR, GS, GOGAT, GDH, NUE, and NAE, while displaying negative correlations with Na^+^/K^+^ ratio, Ss, MDA, O_2_^−^, H_2_O_2_, and PFPN. NUE showed negative correlations with Na^+^/K^+^ ratio, Ss, O_2_^−^, and PFPN, but positive correlations with Proline, H_2_O_2_, POD, CAT, APX, NR, GS, GOGAT and GDH. In the principal component analysis (PCA), PC1 and PC2 of osmoregulatory substances, antioxidant enzymes, nitrogen-metabolizing enzyme activities, and yield in rice contributed 72.0% and 15.0% to the total variance, respectively (**Figure 7**). The results showed that the application of nitrogen fertilizer had effects on Na^+^/K^+^ ratio, physiological indicators, nitrogen-metabolizing enzyme activities if rice in soda saline–alkaline paddy soils.

## 4 Discussion

### 4.1 Nitrogen application significantly improves the physiological status of rice in soda saline-alkaline paddy soils

The high concentration of Na^+^ in soil under saline-alkali stress can disrupt the dynamic ion balance within plant cells, leading to various harmful effects, such as damage to cell membrane structure, disruption of cellular metabolism, and reduced uptake of mineral nutrients and water by the plant (Santos et al., 2021; Teng et al., 2025). A significant influx of Na^+^ into the cytoplasm can lower the membrane potential below its resting level, activating K^+^ efflux channels and disturbing the steady-state equilibrium of the K^+^/Na^+^ ratio under saline-alkali stress (Saifullah et al., 2018; Wang et al., 2024). Consequently, enhancing the equilibrium of sodium and potassium ions within plant tissues is regarded as a viable approach to mitigate saline-alkali stress and promote the reclamation of saline-alkali soils (Chakraborty et al., 2016; Feng et al., 2025). The results of this three-year study demonstrate that the application of N fertilizer following the high-yield and high-efficiency model consistently increased K^+^ concentrations and concomitantly decreased the Na^+^/K^+^ ratio in rice leaves. Additionally, the sodium-potassium ion balance in rice tended to improve linearly with an increase in the nitrogen application rate (**Figure 1**). The positive effect of N fertilizer input, particularly at elevated application rates, on the Na^+^/K^+^ ratio of rice in soda saline-alkaline paddy soils can be attributed to several factors. Firstly, the physicochemical properties of soda saline-alkaline soils result in extremely low nitrogen availability. The application of nitrogen fertilizer significantly promotes root growth in crops and enhances nutrient and water uptake, which subsequently improves the sodium-potassium ion balance and the plants’ ability to withstand stress conditions (Li et al., 2022; Javed et al., 2026). On the other hand, sufficient nitrogen availability modulates root nutrient uptake preferences, favoring ions like K^+^ and Ca^2+^, while simultaneously enhancing the capacity of leaves to exclude Na^+^, thereby strengthening crop resilience to salt stress (Kabir et al., 2005; Jin et al., 2024). Therefore, our research indicates that increasing the input rate of nitrogen fertilizer can reduce the toxic effects of sodium ions and maintain cell activity by maintaining the sodium-potassium ion balance in the organs of rice in soda saline-alkali soils (**Figure 1**). Javed et al. (2021) also found similar results in maize under salinity stress.

Saline-alkaline stress also inhibits plant growth and development through osmotic stress and oxidative stress (Xu et al., 2025). Additionally, the high pH environment of the soil can cause further damage to crops. When the soil pH around the roots increases, certain metal ions such as Fe²^+^, Mg²^+^, and Ca²^+^ tend to precipitate, accompanied by a reduction in inorganic anions (Liang et al., 2024). This impairs the plant’s ability to absorb essential mineral nutrients, leading to severe nutritional stress, which subsequently disrupts various metabolic processes in the plant (Bao et al., 2019; Wang et al., 2023). To counteract saline-alkaline stress, plants mitigate the adverse effects of this stress through mechanisms that include the regulation of osmotic substances, the modulation of ion absorption and transport, and the scavenging of reactive oxygen species (ROS). Previous studies have found that adequate nitrogen application in salt-affected soils can minimize the deleterious effects of salinity on the growth and development of barley (Fayez et al., 2014), and mustard (Rais et al., 2013) by regulating osmotic stress, enhancing antioxidant activities, increasing stomatal conductance, and improving photosynthetic processes. The results of this study demonstrated that over three consecutive years of field experiments, the input of nitrogen fertilizer based on the high-yield and high-efficiency model, particularly at a rate of 275 kg ha^−1^, significantly reduced the levels of MDA (**Figures 2a, b**) and O_2_^−^ (**Figures 2c, d**) in rice leaves during the heading and filling stages under soda saline-alkali paddy soils. Conversely, the contents of soluble protein (**Figure 3b**) and proline (**Figure 3c**) showed significant increases, with the most pronounced enhancement observed in the N4 treatment (275 kg ha^-1^). Further analysis of antioxidant enzymes revealed that the activities of POD (**Figure 4b**), CAT (**Figure 4c**), and ascorbate APX (**Figure 4d**) in rice grown in soda saline-alkaline soils were significantly elevated during both the heading and filling stages. Notably, the highest increases were observed in the N4 (275 kg ha^−1^) and N3 (225 kg ha^−1^) treatments over the three consecutive years of experimentation (2022-2024). In contrast, the activity of SOD (**Figure 4a**) showed the most significant increase in the first two years with the N4 treatment; however, as the growing seasons progressed, the SOD levels in the N5 treatment exhibited a significant decline. In conclusion, the findings of this study indicate that appropriate increasing nitrogen fertilizer input under the high-yield and high-efficiency management strategy effectively enhances the physiological status of rice and mitigates saline-alkali stress in soda saline-alkaline paddy soils. This improvement may be attributed to two main factors: first, the application of nitrogen fertilizer helps to regulate the ion balance in rice organs (**Figure 1**), thereby reducing the toxic effects of Na^+^ and enhancing cellular osmotic regulation, which is crucial for maintaining normal cellular structure and physiological functions (Bao et al., 2019; Piao et al., 2022). Second, the use of nitrogen fertilizer optimizes the nutritional supply to rice, promotes root growth, and stimulates the secretion of organic acids (Javed et al., 2026; Saifullah et al., 2018; Li et al., 2022), thereby enhancing the plant’s adaptability to salt-alkali stress. It is noteworthy that the highest nitrogen application treatment (N5, 325 kg ha^−1^) exhibited a lesser effect in alleviating saline-alkali stress in rice compared to the N4 (275 kg ha^−1^) and N3 (225 kg ha^−1^) treatments. This phenomenon may be attributed to the excessive application of nitrogen fertilizer, which can increase the concentration of salt ions in the soil solution, thereby inhibiting crop growth (Javed et al., 2026). Additionally, Javed et al. (2021) reported that excessive nitrogen application (320 kg N ha^−1^) resulted in a decline in the physiological and biochemical parameters of maize due to nitrogen toxicity in the plants.

### 4.2 Nitrogen application significantly promotes the increase in nitrogen metabolism enzymes activity and nitrogen use efficiency of rice in soda saline-alkaline paddy soils

Soda saline-alkali soils are characterized by high salinity, elevated pH, poor physicochemical properties, and low nutrient availability, which collectively contribute to low nitrogen use efficiency (NUE) in paddy fields (Huang et al., 2010; Gao et al., 2024). When soil pH exceeds 8, nitrification is inhibited, leading to substantial nitrogen loss via volatilization (Borchard et al., 2018). Furthermore, this stress disrupts ionic equilibrium in plant cells, altering proton gradients and impairing NO_3_^−^ uptake (Wang et al., 2012). The competition from HCO_3_^−^ and CO_3_²^−^ ions under such conditions further diminishes NO_3_^−^ concentration and nitrate reductase (NR) activity (Wang et al., 2012; Li et al., 2022). Previous studies have found that increasing the input of nitrogen fertilizer can enhance the saline-alkali resistance of crops by promoting their growth and fertilizer utilization efficiency (Miao et al., 2024; Guo et al., 2021). In this study, we similarly observed that the application of nitrogen fertilizer over three consecutive years in field experiments significantly enhanced the activities of NR, GS, GOGAT, and GDH during the heading and grain filling stages of rice in soda saline-alkali soils (**Figure 5**). Notably, the treatments N4 (275 kg ha^−1^) exhibited the most pronounced effects. Additionally, a significant interactive effect between year and nitrogen fertilizer treatment on the activities of nitrogen metabolism enzymes was identified. Research indicates that plants exhibiting elevated activities of NR and GS, and GOGAT, or those capable of sustaining a higher influx of NO_3_^-^, demonstrate greater tolerance to salt stress (Rais et al., 2013; Liu et al., 2023). Therefore, it is suggested that the application of nitrogen fertilizer in severely soda saline-alkali paddy soils can enhance ionic balance (**Figure 1**), regulate osmotic stress (**Figure 2**), and increase antioxidant activity (**Figure 4**), thereby improving the physiological and nutritional status of the rice (**Figure 3**; Piao et al., 2022; Miao et al., 2024). This, in turn, activates the activities of nitrogen metabolism enzymes (**Figure 5**), ultimately promoting the enhancement of salt and alkali tolerance in rice. The correlation analysis presented in **Figure 6** further supports this conclusion. Additionally, Teh et al. (2016) demonstrated that increasing the activity of nitrogen metabolism enzymes can enhance the tolerance of rice to saline stress. This three-year study also found that an appropriate increase in nitrogen fertilizer under the high-yield and high-efficiency management strategy significantly enhanced nitrogen translocation from stem sheaths and leaves to the panicle from heading to maturity, as well as its contribution to grain nitrogen (**Table 2**). Concurrently, the total N uptake, NUE, and NAE in rice were significantly enhanced (**Table 3**). This improvement can be primarily attributed to the following factors: (i) the application of nitrogen fertilizer significantly alleviated the saline-alkali stress in rice grown in soda saline-alkali paddy soils (**Figures 1**–**4** and **Figure 6**); (ii) it improved the root structure and physiological functions of rice, thereby promoting the absorption of water and nitrogen (Saifullah et al., 2018; Li et al., 2022; Javed et al., 2026); and (iii) nitrogen application markedly enhanced the activity of nitrogen metabolism enzymes in rice (**Figure 5**), thereby facilitating nitrogen absorption and conversion. However, it is noteworthy that the nitrogen translocation amount in stems and leaves, total nitrogen uptake, NUE, and NAE all reached their optimal levels under the N4 (275 kg ha^-1^) and N3 (225 kg ha^-1^) treatments (**Table 3**). This phenomenon may be attributed to the potential nitrogen toxicity effects caused by excessive nitrogen fertilizer application (N5, 325kg ha^-1^) in soda saline-alkali paddy fields, where the applied amount significantly exceeds the nitrogen demand for maximum yield (Javed et al., 2021).

### 4.3 Nitrogen application treatment significantly promotes the increase in the grain yield of in soda saline-alkaline paddy soils

Research indicates that saline-alkali stress induces metabolic disorders in plants through ionic toxicity, osmotic stress, oxidative stress, and high pH stress (Feng et al., 2025). This condition severely inhibits the normal physiological and biochemical processes of crops, as well as their absorption of nitrogen and moisture, leading to a significant reduction in photosynthetic capacity and ultimately resulting in a marked decline in crop yield (Wang et al., 2023; Javed et al., 2021; Song et al., 2022). Research has demonstrated that the practices of additional nitrogen fertilizer in salt-stressed soils can alleviate saline-alkali stress by increasing stomatal conductance, enhancing photosynthetic processes (Nazir et al., 2023), and activating the activities of nitrogen assimilation enzymes (Teh et al., 2016). Furthermore, this practice regulates the selectivity of crop roots for nutrient absorption and improves the ability of leaves to exclude salt ions, ultimately promoting crop tolerance to salt stress and enhancing yield (Teng et al., 2025).

This study demonstrates that the application of additional nitrogen fertilizer under the high-yield and high-efficiency management strategy significantly enhances the grain yield of rice grown in soda saline-alkali soils, with the N4 treatment (275 kg ha^−1^) exhibiting the highest yield increase over three consecutive years of field experiments. Further analysis of the yield components revealed that the increase in the number of panicles per unit area and the number of grains per panicle are the key factors contributing to the enhanced yield of rice (**Table 4**). The results of this study offer a theoretical foundation for alleviating the challenges posed by severe soda saline-alkali soils, enhancing nitrogen use efficiency, and boosting yields in these environments, thereby supporting the sustainable and healthy advancement of paddy rice cultivation in soda saline-alkali regions. The mechanisms that contribute to the efficacy of applying nitrogen fertilizer, especially at a rate of 275 kg ha^−1^ (N4), in enhancing the NUE and yield of rice can be attributed to multiple factors. Firstly, the application of nitrogen fertilizer markedly elevated the potassium ion concentration in the organs of rice cultivated in soda saline-alkali soils and significantly improved the sodium-potassium ion balance (**Figure 1**). This improvement effectively reduces the impacts of ionic toxicity and osmotic stress on the growth and development of rice. Secondly, the application of nitrogen fertilizer significantly mitigated oxidative stress in rice cultivated in soda saline-alkali soils by modulating osmotic regulatory substances (**Figure 3**) and enhancing antioxidant enzyme activities (**Figure 4**). This, in turn, contributed to the preservation of the integrity of cell membrane structures and functions (**Figure 2**). Furthermore, the application of supplemental nitrogen fertilizer aids in maintaining nitrogen balance during the late growth stages, which in turn delays leaf senescence, enhances the photosynthetic rate, and extends the grain filling period (Bao et al., 2019; Javed et al., 2021; Teng et al., 2025). Lastly, the application of nitrogen fertilizer significantly improves total nitrogen uptake (**Table 3**) and the amount of nitrogen translocated (**Table 2**) by enhancing the activity of nitrogen-metabolizing enzymes, particularly NR (**Figure 5a**), GS (**Figure 5b**), and GOGAT (**Figure 5c**). This treatment also increases NUE and NAE (**Table 3**) when compared to the non-nitrogen fertilizer control (N0). The correlation analysis results also confirm this conclusion (**Figures 6**, **7**). Hence, the input of additional nitrogen fertilizer following the high-yield and high-efficiency field management practices, especially at the level of 275 kg ha^−1^, is an important strategy to enhance rice yield and fertilizer utilization efficiency in soda saline-alkali paddy soils.

## 5 Conclusion

This three-year field investigation demonstrated that supplementary nitrogen fertilizer (275 kg ha^−1^) applied based on high-yield and high-efficiency field management practices effectively alleviated ionic toxicity, osmotic and oxidative stress in rice grown in soda saline-alkali paddy soils. This mitigation was evidenced by a reduced leaf Na^+^/K^+^ ratio, lower levels of O_2_^−^ and MDA, and concurrent increases in K^+^ concentration, osmolytes (soluble protein, proline), and antioxidant enzyme (POD, CAT, APX) activities. Furthermore, the input of supplementary nitrogen fertilizer significantly enhanced the activities of enzymes such as NR, GS, GOGAT, and GDH. This enhancement led to an increased translocation amount of nitrogen within the stem sheaths and leaves of rice from the heading stage to maturity. Consequently, there was a notable improvement in the contribution of nitrogen translocation to the panicle, as well as in total nitrogen uptake, NUE, and NAE in rice grown in soda saline-alkali paddy soils. The application of additional nitrogen fertilizer significantly increases rice yield in saline-alkali soils, with the primary contributors to this yield enhancement being the increase in the number of panicles per unit area and the number of grains per panicle. These results underscore the positive and sustainable benefits of an appropriately increased application of nitrogen fertilizer (275 kg ha^−1^) within the high-yield and high-efficiency management practices for alleviating saline-alkali stress, enhancing rice productivity, and improving nitrogen use efficiency in soda saline-alkali paddy soils.

## Data Availability

The raw data supporting the conclusions of this article will be made available by the authors, without undue reservation.
