# Correction: Nitrogen-mediated improvement of ionic homeostasis and antioxidant capacity enhances rice yield and nitrogen use efficiency under soda saline-alkali stress

**DOI:** 10.3389/fpls.2026.1827276

**Published:** 2026-03-26

**Authors:** Frontiers Production Office

**Affiliations:** Frontiers Media SA, Lausanne, Switzerland

**Keywords:** nitrogen metabolism enzyme, nitrogen use efficiency, rice, soda saline-alkali, stress physiology

Affiliation “Gazipur Agricultural University, Bangladesh” of Reviewer Satyen Mondal was erroneously given as “Bangabandhu Sheikh Mujibur Rahman Agricultural University, Bangladesh”.

The original version of this article has been updated.

